# Chiropractic & Osteopathy. A new journal

**DOI:** 10.1186/1746-1340-13-1

**Published:** 2005-04-11

**Authors:** Bruce F Walker, Simon D French, Melainie Cameron

**Affiliations:** 1 Editor-in-Chief, School of Medicine, James Cook University, Townsville, Australia; 2 Associate Editor, Australasian Cochrane Centre, Institute of Health Services Research, Monash University, Clayton, Australia; 3 Associate Editor, School of Health Science, Victoria University, Melbourne, Australia

## Abstract

Both chiropractic and osteopathy are over a century old. They are now regarded as complementary health professions. There is an imperative for both professions to research the principles and claims that underpin them, and the new journal *Chiropractic & Osteopathy *provides a scientific forum for the publication of such research.

## Introduction

In 1959 Frederick George Roberts founded the Chiropractic and Osteopathic College of Australasia (COCA). The Melbourne based College graduated about two hundred chiropractic and osteopathic practitioners from the period 1959 to 1979. The College closed its undergraduate program in 1979 and the students transferred to the Preston Institute of Technology chiropractic program. This is now the Royal Melbourne Institute of Technology (RMIT University) chiropractic course. An osteopathic course commenced alongside the chiropractic course at Phillip Institute of technology (now also RMIT University) in 1986.

Even though it has now closed its undergraduate operations, the College has maintained its company structure and acted as a repository for the records of its alma mater [1].

In 1990 another organisation the Chiropractors and Osteopaths Musculo-Skeletal Interest Group (COMSIG) commenced. Several years later and after steady growth, COMSIG underwent a name change and incorporated under the company structure and banner of the Chiropractic & Osteopathic College of Australasia. From this beginning COCA has grown into the leading provider of post-graduate vocational training for both professions in Australia [1].

In 1992 COMSIG started its own journal and this was known as *COMSIG Review*. In 1995 after incorporation under the COCA banner the journal changed its name to *Australasian Chiropractic & Osteopathy*. It is this journal that has changed from a print journal to the Open Access, online journal *Chiropractic & Osteopathy*.

## The Chiropractic and Osteopathic professions

Both chiropractic and osteopathy are over a century old. They are now regarded as complementary health professions having started their evolution as alternative health groups; this evolution is still underway. There is an imperative for both professions to research the principles and claims that underpin them, and *Chiropractic & Osteopathy *provides a scientific forum for the publication of such research.

For many years both professions were driven by ideology rather than science. This has gradually changed and the pursuit of science is accelerating. The make up of the Editorial Board of *Chiropractic & Osteopathy *reflects this change.

## The Editorial Board

The Editorial Board () is a mix of academics and researchers from a broad cross-section of professions. These include chiropractic, osteopathy, epidemiology, public health, orthopaedics, surgery, rheumatology, biomechanics, education, ergonomics, biostatistics, demography, sociology and radiology.

The main Editorial team (Bruce F. Walker, Simon French, Melainie Cameron, John Jannese and Alan Ralph) take care of the day to day running of the journal.

## Journal content

*Chiropractic & Osteopathy *will encompass all aspects of evidence-based information that is clinically relevant to chiropractors, osteopaths and related health care professionals.

Manuscripts submitted to *Chiropractic & Osteopathy *will initially be reviewed by the Editorial team and subsequently by two external reviewers. Reviewers will be asked to indicate whether the manuscript is scientifically sound, relevant and also to indicate the level of interest to chiropractic and osteopathy professionals.

*Chiropractic & Osteopathy *will consider the following types of manuscripts: primary research, case reports, reviews (both systematic and narrative), commentaries, database articles, debate articles, hypotheses, methodology articles, short reports and study protocols.

The journal has Australian ancestry but now has an international span. As an Open Access journal, *Chiropractic and Osteopathy *will reach a wider audience, enabling the sharing of knowledge with practitioners, researchers and clinicians worldwide. The aim of the journal is to advance the body of chiropractic and osteopathic knowledge.

*Chiropractic & Osteopathy *is published by BioMed Central (), an independent publishing house committed to ensuring that peer-reviewed biomedical research is Open Access – immediately and permanently available online without charge or any other barriers to access.

We at *Chiropractic & Osteopathy *look forward to receiving your submissions.

Researchers interested in submitting a manuscript should see the Instructions to Authors ().

## What is Open Access?

*Chiropractic and Osteopathy's *Open Access policy changes the way articles are published. First, all articles become freely and universally accessible online, and so an author's work can be read by anyone at no cost. Second, the authors hold copyright for their work and grant anyone the right to reproduce and disseminate the article, provided that it is correctly cited and no errors are introduced [2]. Third, a copy of the full text of each Open Access article is permanently archived in an online repository separate from the journal. *Chiropractic and Osteopathy's *articles are archived in PubMed Central [3], the US National Library of Medicine's full-text repository of life science literature, and also in repositories at the University of Potsdam [4] in Germany, at INIST [5] in France and in e-Depot [6], the National Library of the Netherlands' digital archive of all electronic publications.

## The benefits of Open Access

Open Access has four broad benefits for science and the general public. First, authors are assured that their work is disseminated to the widest possible audience, given that there are no barriers to access their work. This is accentuated by the authors being free to reproduce and distribute their work, for example by placing it on their institution's website. It has been suggested that free online articles are more highly cited because of their easier availability [7]. Second, the information available to researchers will not be limited by their library's budget, and the widespread availability of articles will enhance literature searching [8]. Third, the results of publicly funded research will be accessible to all taxpayers and not just those with access to a library with a subscription. As such, Open Access could help to increase public interest in, and support of, research. Note that this public accessibility may become a legal requirement in the USA if the proposed Public Access to Science Act is made law [8]. Fourth, a country's economy will not influence its scientists' ability to access articles because resource-poor countries (and institutions) will be able to read the same material as wealthier ones (although creating access to the internet is another matter [9]).

The Editorial Team look forward to an exciting time ahead for *Chiropractic & Osteopathy*.
